# Recombinant Mammaglobin A Adenovirus-Infected Dendritic Cells Induce Mammaglobin A-Specific CD8^+^ Cytotoxic T Lymphocytes against Breast Cancer Cells In Vitro

**DOI:** 10.1371/journal.pone.0063055

**Published:** 2013-05-01

**Authors:** Huixia Cui, Wenlu Zhang, Wei Hu, Kun Liu, Tong Wang, Nan Ma, Xiaohui Liu, Yunpeng Liu, Youhong Jiang

**Affiliations:** 1 Cancer Research Institute, The First Hospital of China Medical University, Shenyang, China; 2 College of Nursing, Liaoning Medical University, Jinzhou, China; 3 Department of Oncology, The First Hospital of Liaoning Medical University, Jinzhou, China; 4 Department of Medical Oncology, The First Hospital of China Medical University, Shenyang, China; King’s College London, United Kingdom

## Abstract

Mammaglobin A (MGBA) is a novel breast cancer-associated antigen almost exclusively over-expressed in primary and metastatic human breast cancers, making it a potential therapeutic target for breast cancer. The development of dendritic cell (DC)-induced tumor antigen specific CD8^+^ cytotoxic T lymphocytes (CTLs) may hold promise in cancer immunotherapy. In this study we constructed recombinant replication-defective adenoviral (Ad) vectors encoding MGBA and evaluated their ability to trigger anti-tumor immunity in vitro. DCs were isolated from the human peripheral blood monocyte cells (PBMCs) of two HLA-A33^+^ healthy female volunteers, and infected with adenovirus carrying MGBA cDNA (Ad-MGBA). After that, the Ad-MGBA-infected DCs were used to stimulate CD8^+^ CTLs *in vitro* and the latter was used for co-culture with breast cancer cell lines. The data revealed that infection with Ad-MGBA improved DC maturation and up-regulated the expression of co-stimulatory molecules and the secretion of interleukin-12 (IL-12), but down-regulated interleukin-10 (IL-10) secretion from DCs. Ad-MGBA-infected DC-stimulated CD8^+^CTLs displayed the highest cytotoxicity towards HLA-A33^+^/MGBA^+^ breast cancer MDA-MB-415 cells compared with other CD8^+^CTL populations, and compared with the cytotoxicity towards HLA-A33^−^/MGBA^+^ breast cancer HBL-100 cells and HLA-A33^−^/MGBA^−^ breast cancer MDA-MB 231 cells. In addition, Ad-MGBA-infected DC-stimulated CD8^+^ CTLs showed a high level of IFNγ secretion when stimulated with HLA-A33^+^/MGBA^+^ breast cancer MDA-MB-415 cells, but not when stimulated with HLA-A33^−^/MGBA^+^ HBL-100 and HLA-A33^−^/MGBA^−^MDA-MB-231 cells. In addition, killing of CD8^+^CTLs against breast cancer was in a major histocompability complex (MHC)-limited pattern. Finally, the data also determined the importance of TNF-α in activating DCs and T cells. These data together suggest that MGBA recombinant adenovirus-infected DCs could induce specific anti-tumor immunity against MGBA^+^ breast cancers, which could provide a novel strategy in the immunotherapy of breast cancer.

## Introduction

Breast cancer is the most common malignancy affecting women in the world. The worldwide incidence of breast cancer has significantly increased within the past several decades, and in some parts of China, it is ranked to be the most common female invasive cancer [1]. Furthermore, survival rates of breast cancer patients vary greatly depending on cancer type, clinical stage, and treatment. To date, surgery, chemotherapy, and radiotherapy are the major options to treat breast cancer patients, but breast cancer is able to recur. Thus, the exploration of more effective and safer therapeutic modalities is urgently needed. One of these modalities is cancer immunotherapy, which is a growing field of research that studies the body’s immune system in relation to treatment of human cancers.

Dendritic cells (DCs) are the most potent professional antigen-presenting cells (APCs). Once activated, they will process antigen material and present it on the surface to other immune cells in the system [2]–[4]. For example, DCs can present antigen to initial T lymphocytes and in turn activate and change T lymphocytes into antigen-specific cytotoxic T lymphocytes (CTLs) that target tumor cells [5]. Among the activated CTLs, CD8^+^CTLs are believed to be the major effector cells killing target cells [6]–[9]. Furthermore, matured DCs can secrete interleukin-12 (IL-12), whereas reduce production of interleukin-10 (IL-10) that inhibits the CD8^+^T cell immunologic response [10]–[12].

Malignant cells, like breast cancer cells, are derived from mutated autologous normal cells, and may produce specific antigens, so the immune system will develop anti-tumor cell immunogenicity through DC recognition of cancer cell antigens. DCs can enhance antigen-presenting capacity by many means. One of them is pulsing DCs with recombinant and replication-defective adenoviral (Ad) vectors encoding tumor associated antigen, which can elicit antigen-specific CTLs to produce specific anti-tumor effects [3], [13]–[15]. However, the lack of tumor-antigen specificity is the major limitation with cancer immunotherapy. The identification of a cancer antigen, such as a breast cancer specific antigen, is essential for successful cancer immunotherapy. To this end, mammaglobin-A (MGBA), a novel breast cancer-associated antigen, was initially identified using a differential screening approach [16], [17]. The MGBA gene is located on human chromosome 11q13 and is predicted to encode a 10.5 KD protein containing 93 amino acids [17]. The unique property of MGBA is that it is expressed almost exclusively in normal mammary gland epithelium and breast cancer [17], and is overexpressed in up to 80% of primary and metastatic breast cancers [18]–[19]. Although the structure and function of MGBA remains to be defined, its universal expression in breast cancer tissues confirms that it is a promising target for breast cancer immunotherapy [6].

In this study we hypothesized that DCs could present MGBA antigen to activate CD8^+^CTLs, which in turn specifically kill breast cancer cells. We constructed a recombinant adenovirus encoding MGBA to test whether infection could improve DC maturation and whether recombinant adenovirus encoding MGBA infected DCs could elicit potent anti-tumor immune response and lyse breast cancer cells.

## Materials and Methods

### Cell Lines and Culture

Breast cancer MDA-MB-415 and HBL-100 cell lines were purchased from the Type Culture Collection of the Chinese Academy of Sciences, Shanghai, China, while the breast cancer MDA-MB-231 cell line has been maintained in our laboratory for a long time. MDA-MB-415 was cultured in Leibovitz’s L-15 (L15) medium (Gibco, Grand Island, NY) supplemented with 15% defined fetal bovine serum (FBS; Gibco), 10 µg/ml insulin and 10 µg/ml glutathione. MDA-MB-231 was cultured in L15 medium contained 10% FBS, while HBL-100 was cultured in Roswell Park Memorial Institute (RPMI) 1640 medium (HyClone, Logan, UT) containing 10% FBS. All cell lines were maintained at 37°C in a humidified atmosphere of 5% CO_2_ and 95% of air. A series of literature shows the human leucocyte antigen A (HLA-A) genotype of the three cell lines: MDA-MB-415 (HLA-A33^+^), HBL-100 (HLA-A2^+^), and MDA-MB-231 (HLA- A2^+^) [11], [13]–[15].

### Study Subjects

Ethical approval for this study was obtained from our institutional ethics committee (Medical Ethics Committee, The First Hospital of China Medical University). Two healthy female volunteers were enrolled in this study after obtaining their written informed consent. The HLA-A genotype of each subject, examined using sequencing based typing (SBT), was both HLA-A33^+^, which matched that of the breast cancer MDA-MB-415 cell line. The two volunteers were both multipara. One was 34 years old and another was 40 years old.

### Construction of Recombinant Adenovirus Encoding MGBA

We used the pAdxsi Expression System (SinoGenoMax Co Ltd, Beijing, China) to harbor the recombinant MGBA cDNA in the adenovirus vector. Briefly, the full-length MGBA cDNA (coding 93 amino acids protein) was cloned into the shuttle vector pShuttle-CMV and sequenced. Next, it was cloned into the backbone vector pAdxsi to obtain pAdxsi-MGBA plasmid. The desired replication-deficient adenovirus containing MGBA cDNA (Ad-MGBA) was generated by homologous recombination through transfection of the pAdxsi-MGBA plasmid in HEK293 using Lipofectamine 2000 (GIBCO BRL). After several rounds of plaque purification, the adenovirus containing the MGBA gene was amplified and purified from cell lysates by banding twice in CsCl density gradients. Viral products were then aliquoted and stored at −80°C in DMEM containing 2.5% glycerol (v/v) and 2.5% FBS (v/v). The infectious titer was determined by tissue culture infective dose (TCID) 50. The empty virus Ad-null was used as a control.

### Preparation of Dendritic Cells

Peripheral blood mononuclear cells (PBMCs) were isolated from the peripheral blood of the HLA-A33^+^ healthy female volunteers using Ficoll-PaqueTM PLUS (Hao Yang, Tianjin, China). PBMCs were then inoculated in 25 cm^2^ culture flasks at a concentration of 1×10^7^ cells/flask in 5 ml of RPMI-1640 medium (HyClone) containing 10% FBS. After 2 h incubation at 37°C, and 5% CO_2_, the non-adherent cells were removed and the remaining adherent cells were cultured in RPMI-1640 medium supplemented with recombinant granulocyte-macrophage colony stimulating factor (GM-CSF) (1000 IU/ml; R&D, USA) and recombinant interleukin-4 (IL-4) (500 IU/ml; R&D, USA) for 7 days with fresh cytokine medium every 2–3 days. On day 5, the immature DCs were activated by supplementation of tumor necrosis factor α (TNF-α, 1000 IU/ml; PeproTech) in the culture medium. At the end of cell culture (day 7), the mature DCs were harvested for subsequent experiments. During the cultivation, DCs were observed by phase-contrast microscopy and analyzed for surface molecular expression by flow cytometry.

### Adenovirus-mediated MGBA Gene Transfection

On day 5, the immature DCs were collected, counted, and planted in 6-well plates with 1×10^6^ DCs/well in 2 ml of serum-free RPMI 1640 medium. The recombinant adenovirus Ad-MGBA and the empty virus Ad-null were added to the cell culture at a multiplicity of infection (MOI) of 200. After 2 h, 10% FBS was added to the RPMI 1640 medium containing recombinant GM-CSF (1000 IU/ml), IL-4 (500 IU/ml) and TNF-α (1000 IU/ml). The DCs were harvested after 48 h of incubation. MGBA expression in these DCs was determined by Western blotting. IL-10 and IL-12 expression were tested by enzyme-linked immunosorbent assay (ELISA). As control groups, one DC group was added with 50 µg/ml recombinant purified MGBA protein (Abnova, Taiwan) on day 4, and other cytokines were added the same as other DCs. Another DC group was only infected with adenovirus Ad-MGBA without TNF-α to determine the importance of TNF-α in activating DCs and T cells.

### Flow Cytometric Analysis of Gene Expression in DCs

On day 2, 5, and 7, DCs were collected and resuspended in cold phosphate buffered saline (PBS) and then immunostained with a phycoerythrin (PE)-conjugated mouse anti-human CD80, an allophycocyanin (APC)-conjugated mouse anti-human CD83, a fluorescein isothiocyanate (FITC)-conjugated mouse anti-human CD86, or a peridinin-chlorophyll-protein complex (Percp)-conjugated mouse anti-human HLA-DR antibody (BD, USA). The corresponding isotype control antibody (BD) was used as a negative control. To eliminate the effects of different time points, experimental conditions (such as antibody dosage, concentration, incubation durations, cell numbers) were kept consistent. A total of 5×10^5^ cells were incubated with these antibodies at 4°C in the dark for 40 min. These DCs were then washed three times with PBS, resuspended, and analyzed on a FACScan (BD).

### Protein Extraction and Western Blot

The cells were collected and washed twice with PBS, dispersed into a single-cell suspension and homogenized using the Radio Immunoprecipitation Assay (RIPA) lysis buffer (Beyotime, China) on ice. After ultrasonication and high-speed centrifugation, protein concentrations were determined by the bicinchonininc acid (BCA) method. The protein samples were separated by sodium dodecyl sulphate polyacrylamide gel electrophoresis (SDS-PAGE) and then transferred onto a polyvinylidene difluoride (PVDF) membrane. These PVDF membranes were blocked in rabbit serum-PBS. A goat polyclonal anti-human MGBA antibody (Santa Cruz, CA, USA) was used as the primary antibody, and a horseradish peroxidase-conjugated rabbit anti-goat immunoglobulin-G antibody (Amersham Biosciences, UK) was used as the secondary antibody. Immunoreactive bands were detected using the enhanced chemiluminescence (ECL) kit (CWBIO, China).

### Enzyme Linked Immunosorbent Assay (ELISA)

The supernatants from the above 7-day-old DCs (5×10^5^/ml) were collected. Cells and cell debris were then removed through centrifuging. The supernatants were then measured for IL12p70 and IL10 levels by commercial ELISA kits (BioSource) in triplicate according to the manufacturer’s instructions. In particular, 96-well flat-bottom plates were coated with an anti-IL12p70 or IL10 antibody and non-specific binding was blocked by incubation with buffer containing 0.5% BSA. 40 µl cell supernatants, 10 µl antibody and 50 µl biotinylated-horseradish peroxidase (HRP) were added to each well and incubated at 37°C for 60 min. The plates were then washed five times with wash solution, and 50 µl chromogen solutions A and B were added to each well and incubated at 37°C in the dark for 10 min. To terminate the reaction, 50 µl stop buffer was added into each well. The plates were immediately read using a Microplate Reader System (Rayto, China).

### Preparation of MGBA-specifically Stimulated CD8^+^ CTLs

We generated MGBA specifically stimulated CTLs *in vitro* as described previously [3]. Briefly, DCs were transfected with Ad-MGBA or Ad-null at an MOI of 200, and cultured for 2 days in fresh cytokine-supplemented medium. Non-transfected DCs and MGBA protein-added DCs (MGBAp DCs) were used as controls. These DCs were then irradiated with 40 Gy and seeded into 24-well plates at 5×10^4^ cells per well. The non-adherent autologous peripheral blood lymphocytes were added at 1×10^6^ per well. After 7 days of co-culture, the lymphocytes were harvested, resuspended, and then seeded at 5×10^5^ per well. After that, cells were re-stimulated with irradiated DCs at 1×10^5^ per well for 7 days. Then, the cells were again harvested and re-stimulated for the third 7-day period at a ratio of 5∶1 (T:DC). During co-culture, the cells were fed with 50 U/ml of IL-2. On the day 21 day, the CD8^+^ CTLs were purified by selection with Dynabeads® FlowComp™ Human CD8 Kit (Dynal biotech) according to the manufacturer’s instructions, yielding populations consisting of >95% CD8^+^ CTLs (data not shown).

### Detection Cytotoxicity Effects of MGBA Specifically Stimulated CD8^+^ CTLs

The flow cytometry-based CTL assay was used to detect the specific cleaved caspase in target cells according to the modified vendor’s protocol as described previously [20]. Briefly, breast cancer MDA-MB-415, HBL-100, or MDA-MB-231 cell lines were collected, resuspended, and seeded into 6-well plates in 2 ml culture medium at 5×10^4/^well. After 2 h static cultivation, the 1×10^6^ above named CD8^+^ CTLs were added into 6-well plates at ratios of 20∶1 (E:T), which was determined by MDA-MB-415 cells killed by various ratio CD8^+^ CTLs. After 12 h in static incubation, the cell mixture was centrifuged, washed once with PBS, and then immunostained with an Annexin V-PE/7-AAD Apoptosis Detection kit (KeyGen, China) according to the manufacturer’s instructions. The cytotoxicity of MGBA specifically stimulated CD8^+^ CTLs was analyzed by measurement of breast cancer cell apoptosis in the mixture of cells using a FACScan (BD). CD8^+^ CTLs in the mixture were immunostained with a FITC-conjugated mouse anti-human CD8 antibody, so that non-FITC-conjugated breast cancer cells within the mixture were gated to detect apoptosis.

### Immunofluorescence

Immunofluorescence was used to detect apoptosis of breast cancer cells incubated with Ad-MGBA-CD8^+^ CTLs with an Annexin V-FITC/PI Apoptosis Detection kit (KeyGen, China) according to the manufacturer’s instructions. Briefly, breast cancer cells were plated in 6-well chamber cover slides. After 12 h culture, Ad-MGBA-CD8^+^ CTLs was added at ratio of 20∶1 (E:T). After additional co-culture 12 h, the cells on cover slides were washed three times with PBS. Then, 5 µl annexin V-FITC and 5 µl propidium iodide in 500 µl of the binding buffer were added on to cells and incubated for 5 min in the dark. Finally, the apoptosis of breast cancer cells was reviewed under a fluorescence microscope.

### Enzyme linked Immunospot Assay (ELISPOT)

Different CD8^+^ CTLs populations were measured for interferon-γ (IFNγ) secretion by human IFNγ procoated ELISPOT kits (Dakewe, Shenzhen, China) according to the manufacturer’s instructions. Briefly, the plates precoated with anti-IFNγ antibody were incubated with RPMI 1640 medium containing 10% FBS for 10 min at room temperature and then discarded medium. 1×10^5^ CD8^+^ CTLs were co-cultured in triplicate wells with 1×10^3^ breast cancer cells in 100 µl complete medium. CD8^+^ CTLs cultured in the presence of the phytohemagglutinin (PHA; 2.5 µg/mL) were used as a positive control. CD8^+^ CTLs cultured in a complete medium alone were used as a negative control. After 20 h incubation at 37°C and 5% CO_2_, the medium were removed. Cold deionized water was added to the plates to cleave cells at 4°C for 10 min, the plates were washed with 1× washing buffer five times and incubated at 37°C in the presence of biotinylated secondary antibody for 1 h. After five times washes, the plates were incubated at 37°C in the presence of streptavidin-HRP for 1 h and then incubated with the AEC color developing reagent for 30 min. The result spots were counted on a computer assisted ELSPOT image analyzer (Cellular Technology, Ltd., USA). The number of spots observed in the negative control wells was subtracted from the number of spots observed in the experimental wells. Results were expressed as the mean number of IFNγ spots/1×10^5^ cells.

### Statistical Analysis

Data are presented as means ± SD. One-way ANOVA or Student’s t-test was used to determine differences between groups where appropriate. The differences were considered statistically significant when the P value was <0.05.

## Results

### Expression of MGBA Protein in Breast Cancer Cell Lines

**Figure 1:**
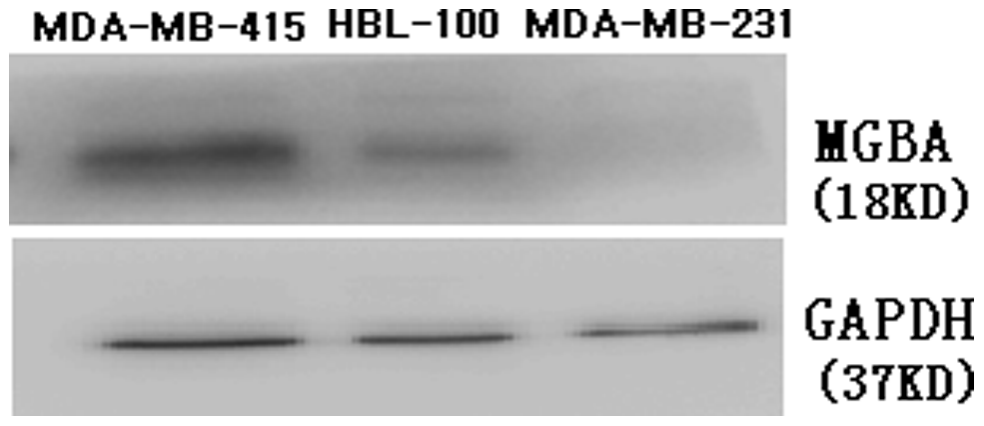
Expression of MGBA protein detected by Western blot in breast cancer cell lines. The 18 KD MGBA protein was detected in MDA-MB-415 and HBL-100, but not in MDA-MB-231.

In this study, we first assessed expression of MGBA protein in these three breast cancer cell lines and found that MGBA protein was detected in MDA-MB-415 and HBL-100, but not in MDA-MB-231 cells (Fig. 1).

### Characteristics of DCs

We isolated DCs from peripheral blood mononuclear cells and then cultured and activated them *in vitro* using recombinant GM-CSF, IL-4, and TNF-α (see Materials and methods section). The morphological characteristics of DCs displayed typical modifications under a phase-contrast microscope from small, round, unequal size and poorly adherent, to large, strongly adherent, cell aggregation and a small quantity of processes at their edges, and to typical morphological characteristics: loosely suspended, irregular cell shapes, fine processes at their edges (data not shown).

**Figure 2:**
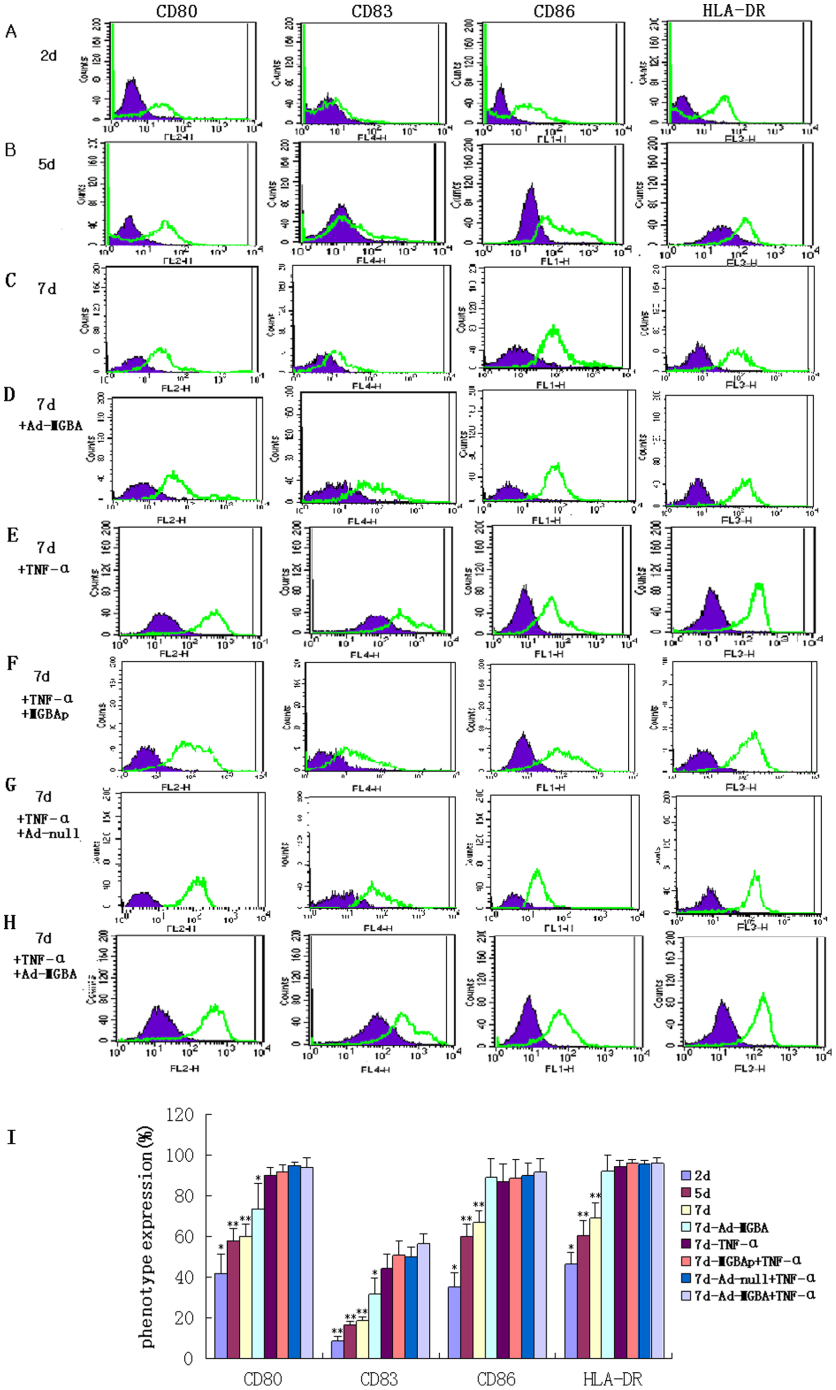
FACS analysis of the changed gene expressions in different DC populations. A-H displays the data of from one volunteer. (A) 2 day culture; (B) 5 day culture; (C) 7 day culture (not stimulated); (D) 7 day culture and 48 h after Ad-MGBA transfection; (E)7 day culture and 48 h after TNF-α addition; (F) 7 day culture, 72 h after recombinant MGBA protein-pulse and 48 h after TNF-α addition; (G) 7 day culture and 48 h after Ad-null transfection and TNF-α addition; (H) 7 day culture and 48 h after Ad-MGBA transfection and TNF-α addition; (I) The histogram shows the data of six independent experiments from two volunteers as mean ± SE. The cultured DCs were collected and stained with PE anti-CD80, APC anti-CD83, FITC anti-CD86, and Percp anti-HLA-DR antibody, respectively. Isotype control for each group is indicated by blue/grey. * indicates that this group has statistically significant differences compared to the others (p<0.05). ** indicates that there are statistically significant differences compared to the groups without ** (p<0.05).

After that, we analyzed the changed gene expression in these DCs using FACS. The data showed that DCs in 2 day culture expressed low levels of CD80, CD83, CD86, and HLA-DR; DCs on day 5 and 7 (not stimulated) expressed modest levels of these proteins, while other DCs on day 7, including 7d-Ad-MGBA DCs, 7d-TNF-α DCs, 7d-MGBAp-TNF-α DCs, 7d-Ad-null-TNF-α DCs and 7d-Ad-MGBA-TNF-α DCs displayed high levels of these proteins (Fig. 2). DCs transfected only with Ad-MGBA for 48 h had a significant increase in these proteins compared with 7d-not-stimulated DCs while they expressed lower CD80 and CD83 than other DCs added TNF-α. 7d-Ad-MGBA-TNF-α DCs expressed the highest levels of these protein in all DCs. The six experiments from two volunteers showed consistent results (Fig. 2I).

### Induction of MGBA Protein Expression in DCs after MGBA-viral Infection

**Figure 3:**
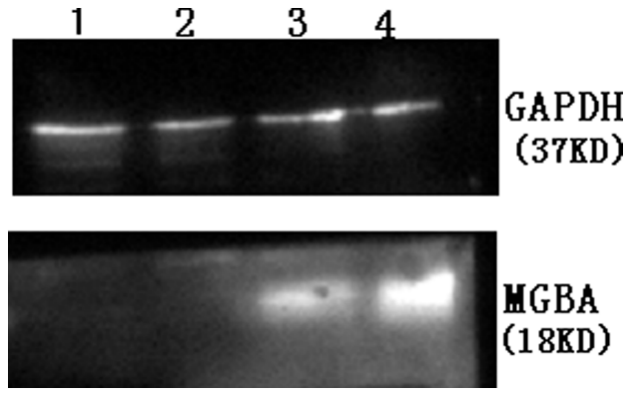
Western blot detection of MGBA protein expression in DCs after Ad-MGBA viral infection. DCs were grown and infected with Ad-MGBA or Ad-null at an MOI of 200 for 48 h. Total cellular protein was extracted and subjected to Western blot analysis of MGBA protein expression. The expression of the MGBA protein was detected in Ad-MGBA-transfected DCs (Lane 4), while there was no expression of the MGBA protein in DCs infected with Ad-null (Lane 1) and non-transfected DCs (Lane 2). Lane 3, MGBA protein expressing MDA-MB-415 cells (as a positive control).

During the DC maturation procedure, we infected the DCs with MGBA virus and, 48 h later, performed a Western blot. Our data showed that expression of MGBA protein was induced in DCs, whereas the Ad-null infected cells or non-transfected DCs did not express any MGBA protein (Fig. 3).

### IL-10 and IL-12 Expression in DCs Infected with Ad-MGBA

**Figure 4:**
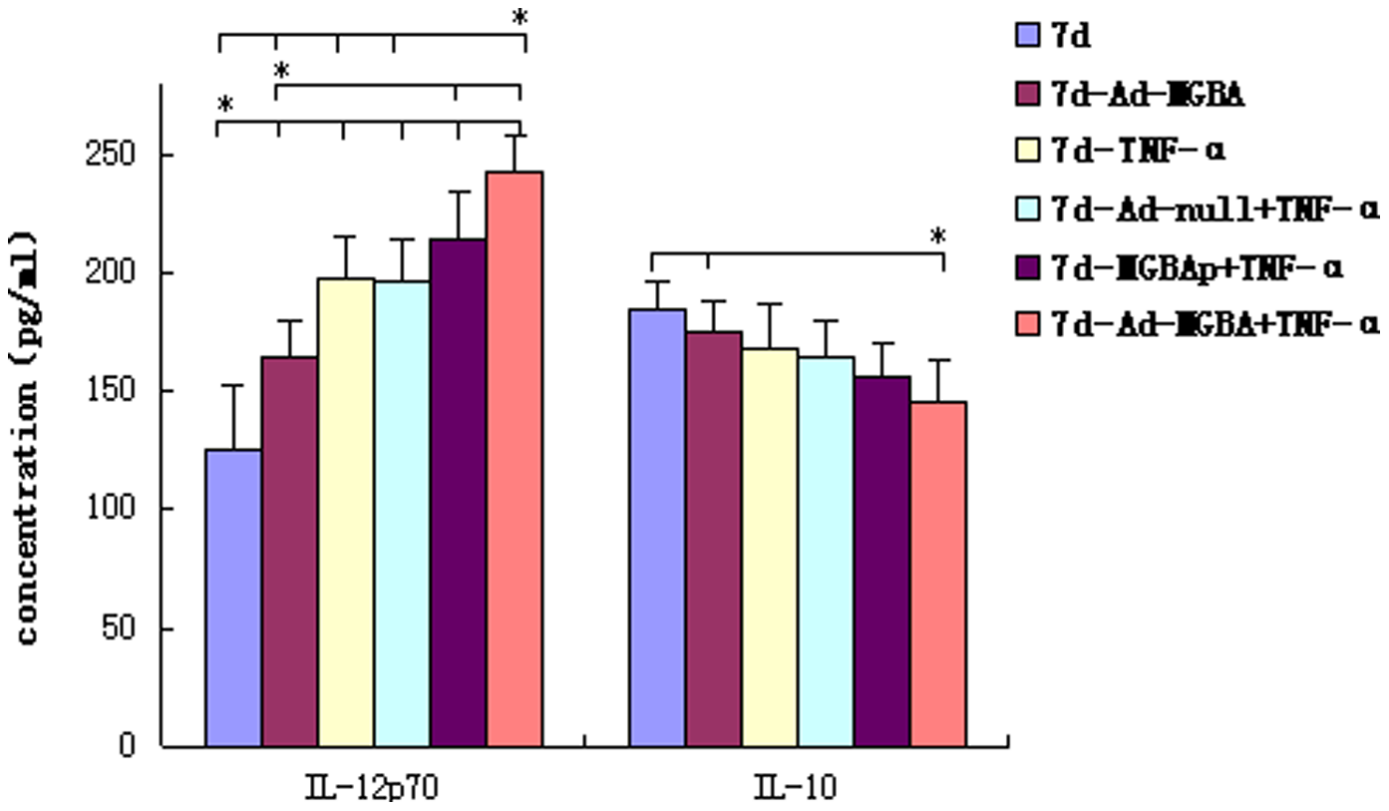
ELISA detection of IL-10 and IL-12 expression in DCs. The supernatants of 7-day-old DCs (5×10^5^/ml) including DCs without any stimulation, DCs transfected only with Ad-MGBA, DCs added with TNF-α, DCs added with Ad-null and TNF-α, DCs added with recombinant MGBA protein and TNF-α, DCs added with Ad-MGBA and TNF-α were collected and assayed in triplicate for levels of IL-10 and IL-12. * indicates that this group has statistically significant differences compared to the others, directed by a short line (p<0.05).

Next, we detected levels of IL-10 and IL-12 expression in DCs using ELISA. As shown in Fig. 4, DCs infected with Ad-MGBA and treated with TNF-α expressed the highest level of IL-12 but the lowest level of IL-10 protein compared with the other DCs. However, the levels of IL-10 and IL-12 expression in DCs infected only with Ad-MGBA were between those of not-stimulated DCs and DCs treated with Ad-MGBA and TNF- α. These data indicate that Ad-MGBA improved DCs to secrete IL-12 but inhibited the secretion of IL-10, suggesting that the immunological reaction was inclined to the TH1 response and that TNF- α addition promoted the procedure.

### MGBA Specifically Stimulated CD8^+^ CTL Cytotoxic Activity *in vitro*

**Figure 5:**
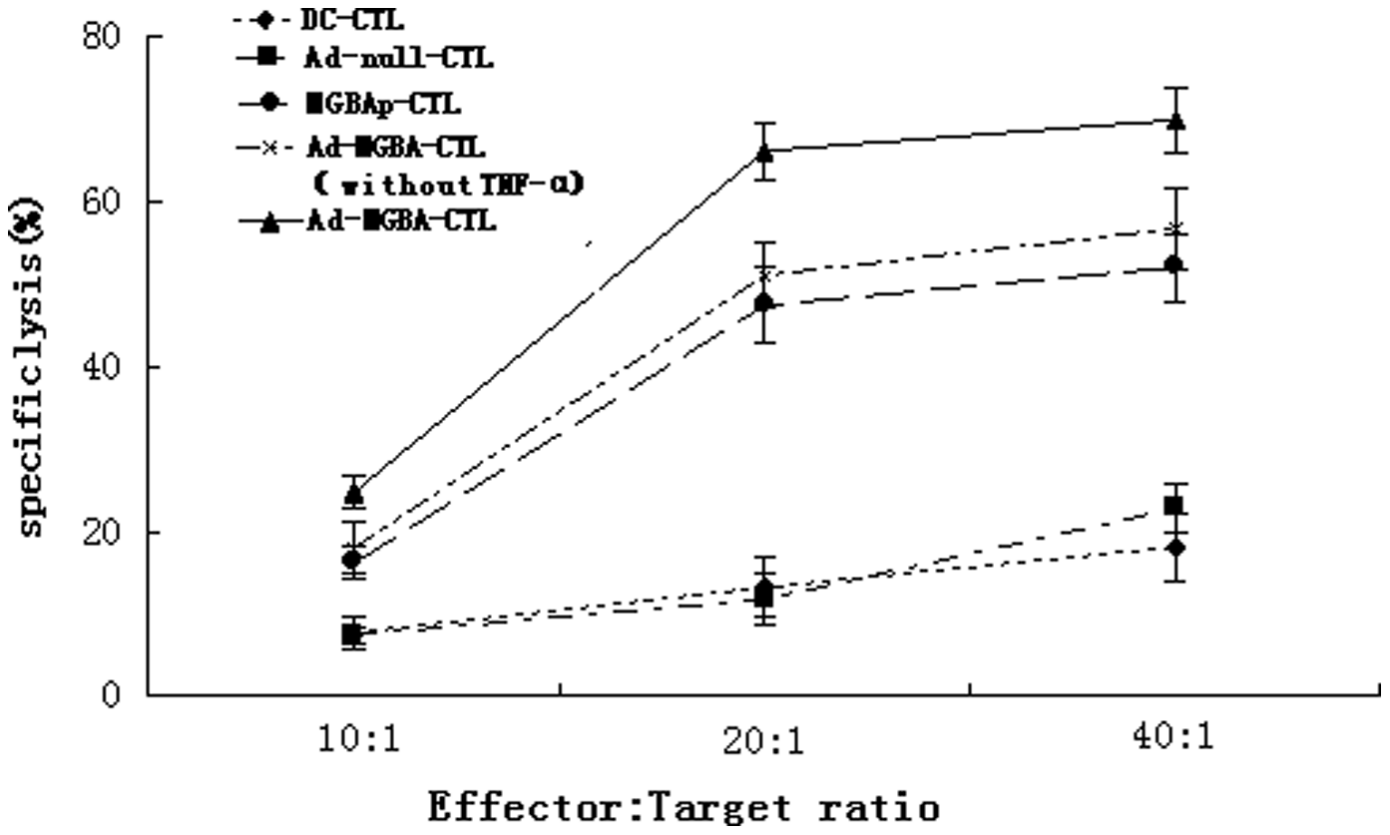
The cytotoxic effects of CD8^+^CTLs against breast cancer MDA-MB-415 cells. The five types of CD8^+^CTLs, i.e., DC-CTL, Ad-null-CTL, MGBAp-CTL, and Ad-MGBA-CTL and Ad-MGBA-CTL (without TNF-α ) were added into MDA-MB-415 cell cultures at an E:T ratio of 10∶1, 20∶1, and 40∶1. 12 h later, breast cancer cell apoptosis rates were analyzed by FACS. The data showed that Ad-MGBA infected DCs-stimulated CD8^+^CTLs had the highest cytotoxic effect among these five types of CD8^+^CTLs. Ad-MGBA-CD8^+^CTL could efficiently lyse MDA-MB-415 cells at an E:T ratio of 20∶1.

**Figure 6:**
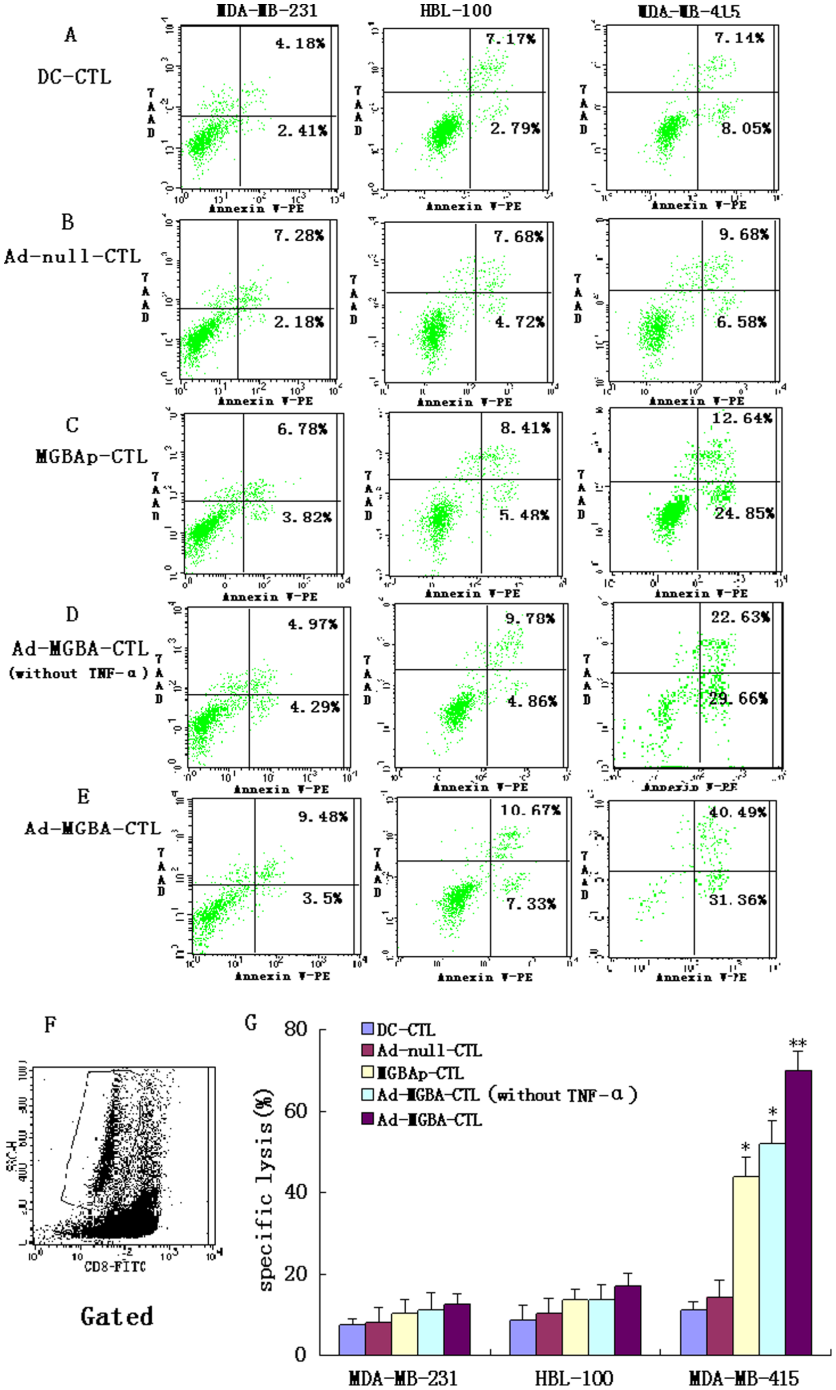
The cytotoxic effects of MGBA specifically stimulated CD8^+^CTLs on breast cancer cells. Four CD8^+^CTLs co-cultured with different DCs treated with TNF-α (i.e., DC-CTL, Ad-null-CTL, MGBAp-CTL, and Ad-MGBA-CTL) and CD8^+^CTLs co-cultured with DCs transfected only with Ad-MGBA (i.e., Ad-MGBA-CTL without TNF-α ) were added into breast cancer cell cultures at an E:T ratio of 20∶1. 12 h later, apoptosis rates of these breast cancer cells were analyzed by FACS. (A) DC-CD8^+^CTLs; (B) Ad-null infected DC-CD8^+^CTLs; (C) MGBAp-CD8^+^CTL; (D) Ad-MGBA infected DC-CD8^+^CTLs (without TNF-α); (E) Ad-MGBA infected DC-CD8^+^CTLs; (F) Non-FITC-CD8-conjugated breast cancer cells in co-culture cells gated; (G) Histogram summarizing the data from six independent experiments from two volunteers. The apoptosis rate in HLA-A33^+^/MGBA^+^ MDA-MB 415 cells induced by Ad-MGBA-CD8^+^CTL was the highest among these five CTLs (p<0.01). * indicates that this group has statistically significant differences compared to the others (p<0.05). ** indicates that this group has statistically significant differences compared to the others (p<0.01).

**Figure 7:**
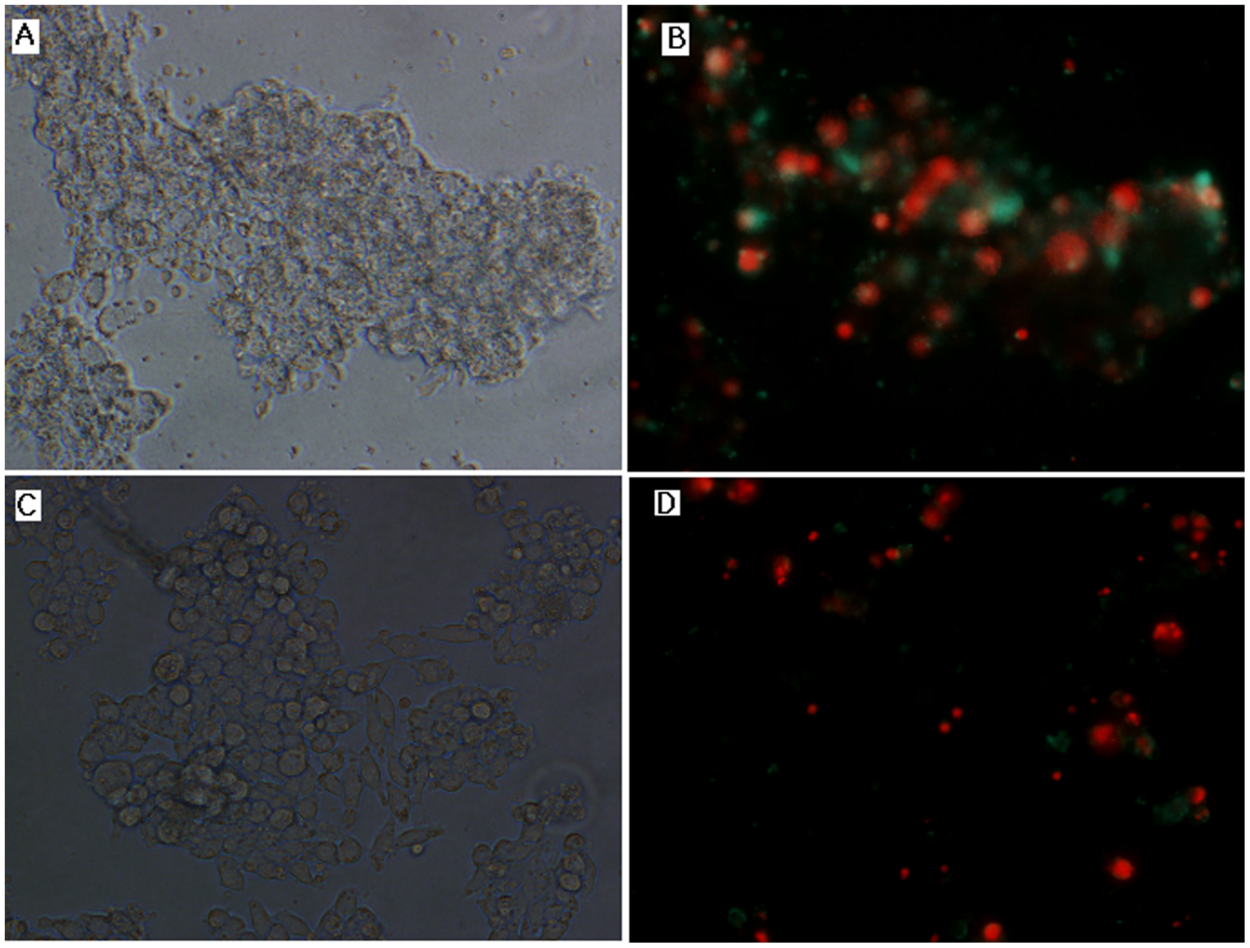
The cytotoxic effects of MGBA specifically stimulated CD8^+^CTLs on breast cancer MDA-MB 415 cells and MDA-MB 231 cells by a fluorescence microscope(x 400). The Ad-MGBA-CD8^+^CTLs were added into breast cancer MDA-MB 415 cell and MDA-MB 231 cells culture at an E:T ratio of 20∶1. 12 h later, the most of MDA-MB 415 cells appeared apoptosis (B, green color) and necrosis (B, red color) while only a few MDA-MB 231 cells appeared apoptosis and necrosis (D). (A) MDA-MB 415 cells on bright field; (B) MDA-MB 415 cells on fluorescence field; (C) MDA-MB 231 cells on bright field; (D) MDA-MB 231 cells on fluorescence field.

DCs from the HLA-A33^+^ donors were isolated and infected with Ad-MGBA or Ad-null vector at an MOI of 200 or pulsed with recombinant MGBA protein. Meanwhile, CTLs were isolated from peripheral blood mononuclear cells and then stimulated with the above DCs for 21 days. After that, four CD8^+^CTLs co-cultured with different DCs treated with TNF-α (i.e., DC-CTL, Ad-null-CTL, MGBAp-CTL, and Ad-MGBA-CTL) and CD8^+^CTLs co-cultured with DCs transfected only with Ad-MGBA (i.e., Ad-MGBA-CTL without TNF-α ) were isolated by using the magnetic beads positive selection from non-adherent peripheral blood lymphocytes. Next, we tested the cytotoxic activity of these CD8^+^CTLs against breast cancer cells. Ad-MGBA-CD8^+^CTLs had the highest cytotoxic activity among the five different kinds of CD8^+^CTLs (Fig. 5). Ad-MGBA-CD8^+^CTLs could efficiently lyse MDA-MB-415 cells at an E:T ratio of 20∶1 because the MDA-MB-415 breast cancer cell apoptosis rate was increased a little at an E:T ratio of 40∶1. Ad-MGBA-CD8^+^CTLs, Ad-MGBA-CD8^+^CTLs (without TNF-α ) and MGBAp-CD8^+^CTLs were able to cause 70.45%, 51.67% and 44.03% of HLA-A33^+^/MGBA^+^ MDA-MB 415 cell lysis, respectively, at an E:T ratio of 20∶1, whereas DC-CD8^+^CTLs and Ad-null-CD8^+^CTLs only caused 11.14% and 14.40% of the cell lysis, respectively (Fig. 6). This result indicated that Ad-MGBA transfection and protein pulse of MGBA could both produce MGBA-specific CD8^+^CTLs, while Ad-MGBA transfection was more efficient than the MGBA protein pulse (p<0.05). Notably, Ad-MGBA-CD8^+^CTLs (without TNF-α ) caused a lower lysis rate of MDA-MB 415 cells than that of Ad-MGBA-CD8^+^CTLs (p<0.01), suggesting the importance of TNF-α in activating T cells. Furthermore, Ad-MGBA-CD8^+^CTLs only caused 17.01% of HLA-A33^−^/MGBA^+^ HBL-100 cell lysis and 12.65% of HLA-A33^−^/MGBA^−^ MDA-MB 231 cell lysis, compared with 70.45% of HLA-A33^+^/MGBA^+^ MDA-MB 415 cell lysis (p<0.01). This result indicated that MGBA specifically stimulated CD8^+^ CTL cytotoxic activity was limited by the major histocompability complex (MHC). Differences among these three breast cancer cells killed by Ad-null CD8^+^ CTLs and DC-CD8^+^ CTLs were not significant (p>0.05). In addition, most of MDA-MB 415 cells incubated with Ad-MGBA-CD8^+^CTLs for 12 h had undergone apoptosis or necrosis, while only a few MDA-MB-231 cells appeared apoptotic (Fig. 7). Taken altogether, these data indicate that Ad-MGBA-infected DCs were able to specifically induce CD8^+^CTLs against HLA-A33-matched and MGBA-positive breast cancer cells.

### Induction of IFNγ Secretion in MGBA-specifically Stimulated CD8^+^CTLs

**Figure 8:**
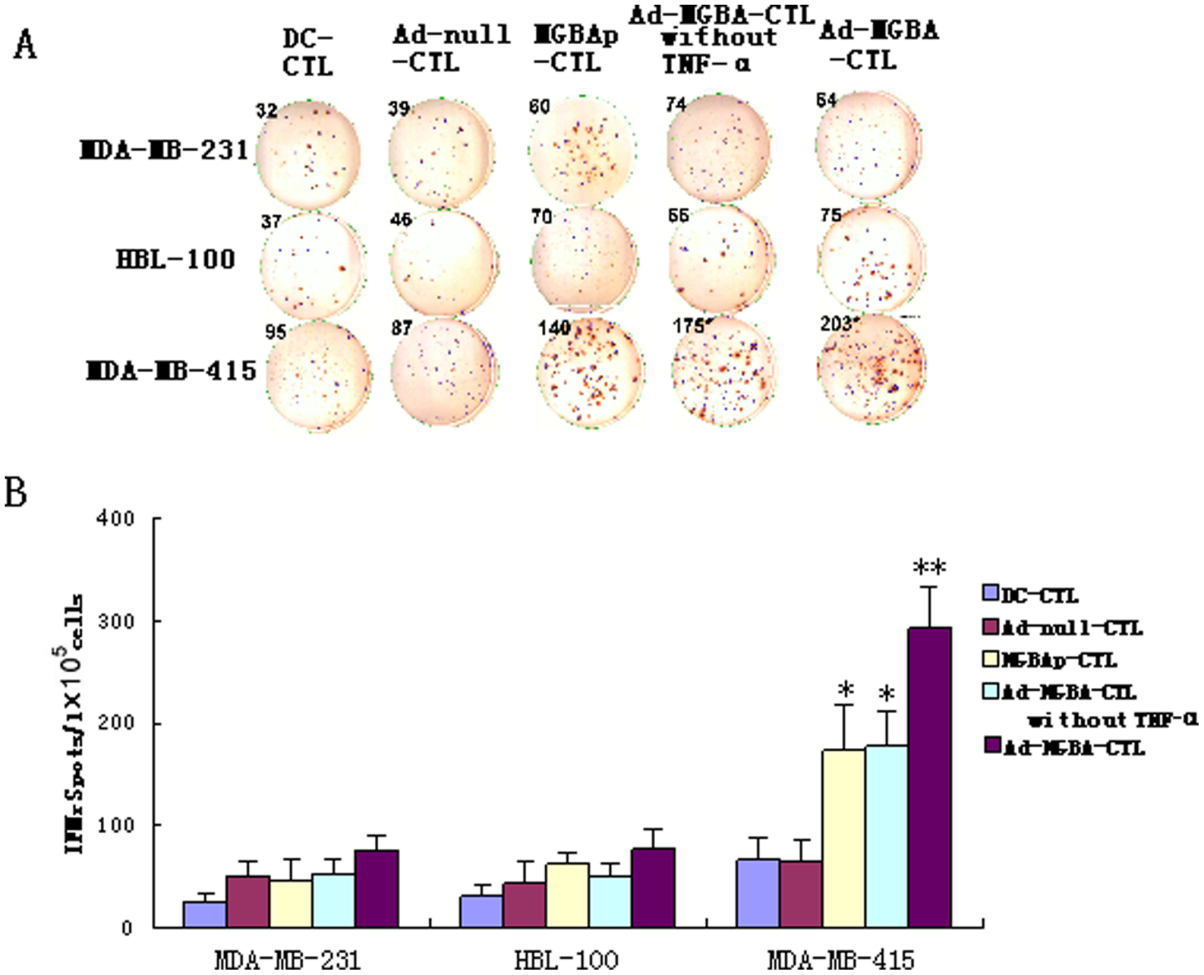
Different CD8^+^ CTL populations were measured for interferon-γ (IFNγ) produce when stimulated with three breast cancer cells by ELISPOT assay. 1×10^5^ CD8^+^ CTLs were cultured in triplicate wells in 100 µl of complete medium containing 1×10^3^ breast cancer cells in 96-well anti-IFNγ antibody precoated plates. After 20 h incubation, IFNγ spots were analyzed. (A) displayed partly spots from a experiment; (B) The histogram shows the data of six independent experiments from two volunteers as mean ± SE. **indicates that this group has statistically significant differences compared to the others (p<0.01). * indicates that there are statistically significant differences compared to the groups without * (p<0.01).

Using the ELISPOT assay, we also detected IFNγ secretion in different CD8^+^CTLs when stimulated with three different breast cancer cell lines. As shown in Fig. 8, Ad-MGBA-CD8^+^CTLs displayed the highest level of IFNγ during killing of MDA-MB-415 cells when compared with the other four CD8^+^CTL populations or when compared with the other two breast cancer cell lines. This result further verified that the cytotoxic activity of Ad-MGBA-CD8^+^CTLs was the highest and was limited by MHC. Furthermore, Ad-MGBA-CD8^+^CTLs (without TNF-α) produced a lower level of IFNγ than Ad-MGBA-CD8^+^CTLs (p<0.01), also suggesting the importance of TNF-α in activating T cells.

## Discussion

### Expression of MGBA Protein in Breast Cancer Cell Lines

In this study, we first analyzed expression of MGBA protein in three breast cancer cell lines and found the MGBA protein was expressed in MDA-MB-415 and HBL-100 with the molecular weight of 18 KD, but not in MDA-MB-231 cells. However, previous studies either predicted it to be 10.5 KD [17] or identified it to be 21 KD and 14 KD using Western blots [21]. MGBA belongs to the secretoglobin gene family that is glycosylated and different levels of glycosylation may change the molecular weight of the protein since MGBA has two consensus N-glycosylation sites [21]. Another reason might be because it may represent incompletely reduced native MGBA [21]. To date, it is unknown whether glycosylation at these sites could affect the function of MGBA protein. Another argument is that MDA-MB-231 cells do not express MGBA protein. MDA-MB-231 cells do not express MGBA protein in our hands, and are known to be ER-negative [22]. Previous studies showing that MGBA expression was associated with the absence of tumor estrogen receptors (ER) [23], [24], but another study does not support these findings [25]. Furthermore, the structure and function of MGBA remain to be undefined [6]. Thus, conclusions with regards to MGBA and ER expression and much unknown information about MGBA need further evaluations.

### Effects of Ad-MGBA Infection on Induction of DCs Maturation and Activation

Next, we successfully isolated DCs from the peripheral blood of the two HLA-A33^+^ healthy female volunteers *in vitro*. After that, we stimulated these cultured DCs using different cytokines (see methods section) and with Ad-MGBA infection or recombinant MGBA protein. Morphologically, the matured DCs showed size and shape changes in DCs. At molecular levels, these matured DCs added TNF-α (Fig.2.E, F, G&H) expressed high levels of different cell surface proteins, such as CD80, DC83, CD86, and MHCII molecule HLA-DR, and 7d-Ad-MGBA-TNF-α DCs expressed the highest levels of these proteins in all DCs. DCs transfected only with Ad-MGBA expressed greater levels of these proteins than 7-day-old non-simulated DCs (p<0.05), suggesting Ad-MGBA could improve DC maturation and activation. However, DCs transfected only with Ad-MGBA expressed lower levels of CD80 and CD83 proteins than DCs treated with TNF-α (p<0.05), suggesting Ad-MGBA could not completely improve DC maturation and activation.

Activated DCs can secrete many cytokines, some of which could mediate Th1 type responses beneficial to activate CD8^+^T cells, whereas others belonged to inhibitory cell factors to induce immunotolerance [12]. For example, IL-12 plays a key role in the initiation of protective innate and adaptive immune responses against intracellular pathogens and also induces the expression of other cytokines, particularly IFNγ [26]. In the immunological reaction, IL-12 promotes development of TH1 type reaction that stimulates cell immunological reaction beneficial to killing tumor cells. In contrast, IL-10 is an inhibitory factor for initial T lymphocyte and TH1 type reaction [10], [27]. In this study, we found that 7d-Ad-MGBA-TNF-α DCs displayed the highest level of IL-12 and the lowest level of IL-10 in all DCs, while 7d-Ad-MGBA DCs without TNF-α expressed moderate levels of IL-12 and IL-10 between those of 7-day-old non-simulated DCs and 7d-Ad-MGBA-TNF-α DCs (p<0.05) (Fig.4). These data suggest that Ad-MGBA infection and TNF-α addition was beneficial to DCs in activation CD8^+^T cells.

*In vitro*, DCs can be generated from peripheral blood monocytes cultured with GM-CSF and IL-4. However, DCs can be further induced to maturity by danger mediators such as bacterial lipopolysaccharides (LPS) or proinflammatory cytokines, mainly TNF-α [28]–[30]. The mechanisms of DC maturation are very complicated and not completely defined. It has been reported that TNF-α can activate MAPK and PI3K signaling pathways via TNFR1, which improve the expression of cell surface molecules and the secretion of cytokines, so that DCs are induced to maturation [31]. In this study, we found the transfection only with Adenovirus could also enhance DC maturation and modulate cytokine production, which were consistent with other studies [32]–[35]. It is reported that this mechanism may be independent of transgene expression, but dependent on viral entry into the cell and/or translocation to the nuclei [36]. It is further shown that it may be adenovirus capsid protein penton that mediates NF-κB activation [37], [38], and activated NF-κB up-regulates a large number of gene expression involved in the immune response, including TNF-α, which in turn further improves DC maturation through an autocrine pathway [37], [39]. However, in this present study, we found the expression levels of cell surface molecules and cytokines of DCs that were only transfected with adenovirus were intermediate between immature and mature DCs. Therefore, exogenous TNF-α was still needed to completely improve DC maturation.

### Ad-MGBA-infected DC Stimulation of CD8^+^CTLs for their Antitumor Activity

Antigen-specific CTLs activated by DCs were always a promising immunotherapeutic strategy against various cancers [10], [40], [41]. However, the interactions between DCs and T lymphocytes are very complicated and may involve different mechanisms [42], [43]. Generally, induction of T lymphocyte activation, proliferation and function not only requires the recognition between the T-cell receptor (TCR) and specific major histocomptability complex (MHC) peptide complexes expressed on the surface of antigen presenting cells (APCs) (such as DCs and tumor cells), but also the interaction between co-stimulatory molecules on T-cells and APCs [44], [45]. Nevertheless, the recognition between TCR of T lymphocytes and antigen presented by DCs was limited by MHC molecules and thus, killing target cells by the activated CTLs was also limited by MHC molecules. Indeed, in our current study, we showed that CD8^+^CTLs were successfully induced by Ad-MGBA-infected DCs (Fig. 6). Although CD8^+^CTLs stimulated with MGBA protein-pulsed DCs could efficiently lyse MDA-MB 415 cells, CD8^+^CTLs stimulated by Ad-MGBA infected DCs with or without added TNF-α had better cytotoxic activity against breast cancer cells compared to the others. In addition, killing occurred mostly in HLA-A33^+^/MGBA^+^ MDA-MB 415 cells, but not in HLA-A33^−^/MGBA^+^ HBL-100 cells and HLA-A33^−^/MGBA^−^ MDA-MB 231 cells, suggesting the antitumor activity of these CD8^+^CTLs is quite specific to the MHC-specific pattern. The current data suggest that use of CTLs to treat cancer patients has to match the HLA types to that of donors. Notably, the CD8^+^CTLs stimulated by Ad-MGBA-infected DCs without TNF-α had lower cytotoxic activity to MDA-MB-415 cells than that of cells stimulated by Ad-MGBA-infected DCs with TNF-α, but had higher cytotoxic activity than that of cells stimulated by MGBA protein-pulsed DCs (p<0.01). These results indicated Ad-MGBA-infected DCs could efficiently induce MGBA-specific CD8^+^CTLs and exogenous TNF-α addition could further enhance the function of these DCs. Moreover, the data on IFNγ expression from co-culture of CTLs and breast cancer cells also confirmed that CD8^+^CTLs stimulated by Ad-MGBA-infected DCs with TNF-α had the highest cytotoxic activity, indicating that this approach could be useful and further developed for breast cancer immunotherapy in the future. Before this, we have to perform *in vivo* animal studies to verify our current data.

This study has verified HLA-A33-limited cytotoxic activity against MGBA antigen, and next, we will continue to identify HLA-A33-binding specific epitopes of MGBA. Of course, besides recombinant MGBA adenovirus transfection-induced antigen-specific CD8^+^CTLs, it may induce antigen-specific CD4^+^ T cells, or induce protective antibodies *in vivo*. Probably, it is also able to induce harmful immune responses to viral proteins *in vivo*. Thus, more experiments are needed to define these in our future study.

### Conclusion

This study demonstrated dendritic cells infected with mammaglobin-A recombinant adenovirus specifically elicited mammaglobin-A specifically stimulated CD8^+^ cytotoxic T lymphocytes against HLA-A33^+^/MGBA^+^ breast cancer cells *in vitro*, suggesting that it may be further developed as a novel strategy for immunotherapy of MGBA-positive breast cancers.
